# Health‐related quality of life and patient‐reported outcome measurements in patients with cystinosis

**DOI:** 10.1002/jmd2.12352

**Published:** 2022-12-16

**Authors:** Stefanie Witt, Kaja Kristensen, Katharina Hohenfellner, Julia Quitmann

**Affiliations:** ^1^ Center for Psychosocial Medicine, Department of Medical Psychology University Medical Center Hamburg‐Eppendorf Hamburg Germany; ^2^ Pediatric Nephrology RoMed Kliniken Rosenheim Germany

**Keywords:** cystinosis, health‐related quality of life, patient‐reported outcome measurements, rare diseases, scoping review

## Abstract

Nephropathic cystinosis is a rare autosomal recessive lysosomal storage disorder. With the availability of treatment and renal replacement therapy, nephropathic cystinosis has evolved from an early fatal disease to a chronic, progressive disorder with potentially high impairment. We aim to review the literature on the health‐related quality of life and identify appropriate patient‐reported outcome measurements to assess the health‐related quality of life of patients with cystinosis. For this review, we conducted a literature search in PubMed and Web of Science in September 2021. Inclusion and exclusion criteria for the selection of articles were defined a priori. We identified 668 unique articles through the search and screened them based on title and abstract. The full texts of 27 articles were assessed. Finally, we included five articles (published between 2009 and 2020) describing the health‐related quality of life in patients with cystinosis. All studies, apart from one, were conducted in the United States, and no condition‐specific measurement was used. Patients with cystinosis reported a lower health‐related quality of life (for certain dimensions) than healthy subjects. Few published studies address the health‐related quality of life of patients with cystinosis. Such data must be collected standardized and follow the FAIR (Findable, Accessible, Interoperable, and Reusable) principles. To gain a comprehensive understanding of the impact of this disorder on health‐related quality of life, it is necessary to use generic and condition‐specific instruments to measure this, preferably in large samples from longitudinal studies. A cystinosis‐specific instrument for measuring health‐related quality of life has yet to be developed.

**Synopsis**

Health‐related quality of life in patients with cystinosis is an under‐researched area that could be imrproved through validated patient‐reported outcomes and qualitative research techniques.

## 1 INTRODUCTION

Nephropathic cystinosis is a rare lysosomal storage disorder with an incidence of 1 in 100 000–200 000 live births and is caused by mutations in the *CTNS* gene, which encodes the protein cystinosin.^1^ When cystinosin is absent or dysfunctional, cystine accumulates within the lysosome as the transportation out of the lysosome is impaired. Intralysosomal cystine accumulation gradually damages cells and tissues of nearly all organs.^2^ If left untreated, the disease progresses steadily and results in end‐stage renal failure by early school age.^1^

Cystinosis is one of the few rare genetic diseases for which treatment is available.^3^ Cysteamine effectively targets intralysosomal accumulation of cystine by reducing it to form cysteine plus a mixed cysteamine–cysteine disulfide that can exit the lysosome via other transporters.^3^ Compliance with cysteamine treatment is challenging for patients due to various therapeutic side effects, including bad breath (halitosis) and gastrointestinal disturbance, in addition to a potentially arduous treatment regimen.^4^

Patients with cystinosis are typically diagnosed at 12–24 months of age (mean 14 months), presenting with stunted growth, rickets, polydipsia, and polyuria resulting from proximal tubular impairment.^5^ The need to replenish electrolytes, correct acid–base imbalance, and initiate cysteamine therapy pose significant challenges for patients and families.^6^

Early diagnosis and treatment positively influence the clinical outcome of patients with nephropathic cystinosis.^7^ As patients survive longer on cysteamine treatment and renal replacement therapy, other functional changes commonly develop, affecting the eyes, muscles, endocrine organs, and central nervous system.^8^ Late complications in patients occur less frequently and at a later stage when patients are treated early and consistently with cysteamine.^1^

Patient survival into adulthood as a result of cysteamine treatment has allowed for the emergence of chronic disease effects within multiple organ systems of the body.^9^ Successful patient transition to adult medical care is a priority for research and clinical practice.

The myriad of disease‐related health effects combined with a demanding treatment regimen can negatively impact the health‐related quality of life (HrQoL) of patients with cystinosis.^10^ HrQoL is a subjective, self‐assessed, and multidimensional construct^11^; it represents physical, emotional, and social well‐being, role functioning, and potentially other domains relevant to the disease being investigated, all from the patient's perspective.^12^

This article reviews the literature on HrQoL in patients with cystinosis intending to improve overall understanding of the subjective experience of living with this rare chronic disorder. On the one hand, we aim to describe HrQoL in patients with cystinosis. On the other hand, we want to identify validated patient‐reported outcome measures (PROMs) that capture HrQoL in this population appropriate for use in research and clinical daily routine.

## 2 MATERIALS AND METHODS

### 2.1 Search strategy

We searched the Web of Science and PubMed databases for relevant literature on 22 September 2021 and updated results as of 07 April 2022. The methodological framework of Arksey and O'Malley's five‐stage scoping review guided the literature review.^13^

We considered qualitative and quantitative empirical studies focusing on HrQoL in patients with cystinosis. For a quantitative study to be considered for inclusion, HrQoL must have explicitly been measured using validated PROMs, along with details on how it was measured. All empirical study design methods were eligible for inclusion (cross‐sectional, cohort, clinical trials, registry, case reports, and case series), while reviews were excluded. There were no geographic, language, or temporal restrictions. We only considered studies published in peer‐reviewed journals, although this included published conference abstracts due to the assumed scarcity of studies. These inclusion and exclusion criteria were agreed upon *a priori*.

As additional constructs, such as psychosocial health, mental health, and well‐being, are sometimes used as a synonym for HrQoL,^14^, ^15^ these terms were included in our search string. The search contained a combination of keywords and MeSH terms: (“Lysosomal Storage Diseases”[Title/Abstract] OR “Lysosomal Storage Diseases”[MeSH] OR “Cystinosis”[Title/Abstract] OR Cystinosis[MeSH] OR “Fanconi syndrome”[Title/Abstract] OR “Nephropathic Cystinosis”[Title/Abstract]) AND (“quality of life”[Title/Abstract] OR “well‐being”[Title/Abstract] OR “Psychosocial health”[Title/Abstract] OR “mental health”[Title/Abstract] OR “quality of life”[MeSH]).

### 2.2 Selection process

After using the agreed search terms in the databases and removing duplicated publications, two independent reviewers screened the search results based on the title and abstract. The same two reviewers thoroughly read the full texts of any articles that met the inclusion criteria or for which it was not possible to assess eligibility based on the title and abstract alone, with subsequent evaluation for eligibility. The reviewers discussed any discrepancies or ambiguities regarding inclusion eligibility until a consensus was reached. In addition, reference lists of included publications were also reviewed for possible inclusion using the same eligibility criteria.

### 2.3 Data extraction

Data on the author, year of publication, country of origin, study objective, study design, number of participants, participant characteristics, HrQoL measure, and the main study results were recorded as well as PROMs' characteristics (e.g., scales, number of items, reliability, languages, and age groups).

## 3 RESULTS

### 3.1 Study selection

**FIGURE 1:**
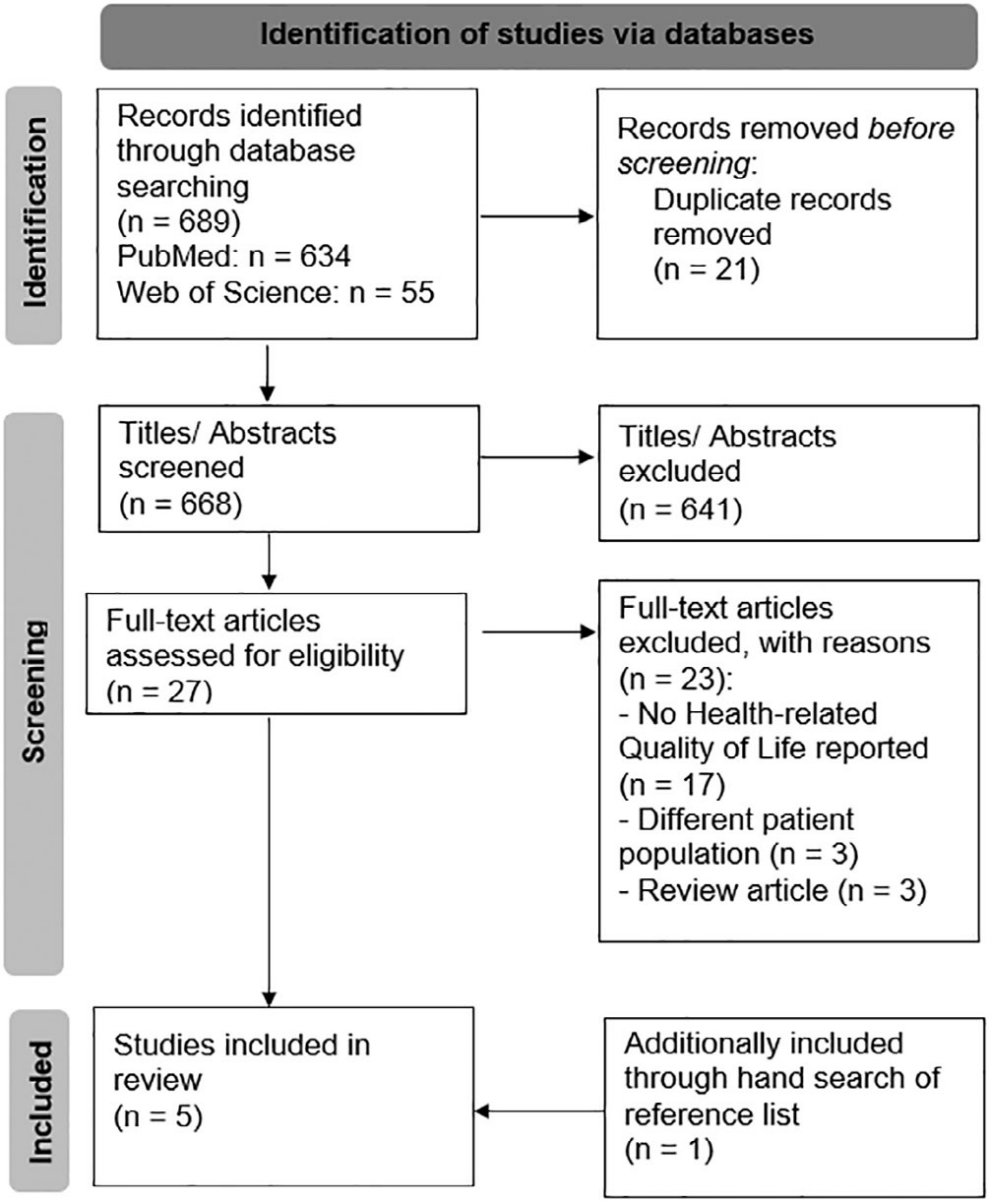
PRISMA flowchart of the study selection process

The search terms identified 668 unique publications after removing any duplicates. Based on the title and abstract screening, 641 publications were excluded, while 27 publications were selected for full‐text screening. Of these, four publications met the inclusion criteria.^10^, ^16^, ^17^, ^18^ A review of the references for these four publications yielded one additional relevant paper,^19^ resulting in five eligible studies. Figure 1, based on the PRISMA Statement,^20^ depicts a flow diagram of the search process and respective outputs.

### 3.2 Study characteristics

Table 1 briefly summarizes the five included studies.^10^, ^16^, ^17^, ^18^, ^19^ These studies were published between 2009 and 2020. All studies were conducted in the United States, except one from Switzerland.^10^ Study designs varied widely and included case series,^10^ cross‐sectional,^16^ qualitative,^19^ and prospective^17^, ^18^ study designs. Four studies assessed HrQoL using PROMs.^10^, ^16^, ^17^, ^18^ In these studies, two generic instruments (Pediatric Quality of Life Inventory (PedsQL),^16^, ^17^ The Netherlands Organization for Applied Scientific Research Academical Medical Center (TNO–AZL) Child Quality of Life Questionnaire (TACQOL)),^10^ and one condition‐specific instruments (Monroe Dunaway Anderson Dysphagia Inventory (MDADI))^18^ were used. One study did not use a quantitative PROM to measure HrQoL, instead exploring qualitatively how patients with cystinosis experience their transition to adulthood.^19^

**TABLE 1:** Summary of included studies—HrQoL in patients with cystinosis

Authors (year)	Country of origin	Objective	Study design	Sample size, n	Sample description	HrQoL assessment and analysis	Results
Ulmer et al. (2009)^10^	Switzerland	To assess intellectual and motor performance, QoL, and psychosocial adjustment in children and adolescents with cystinosis	Case series	9	4 female, 5 male age range: 5–19 years	TACQOL parent and child version (data comparison with reference values)	Child‐reported QoL was normal for six of seven dimensions [exception: positive emotions (10.9 vs. 13.3; *p* ≤ 0.05)]. Parent‐reported QoL was impaired in four dimensions: positive emotions (11.4 vs. 15.0; *p* ≤ 0.01), autonomy (27.0 vs. 31.4; *p* ≤ 0.05), social function (26.0 vs. 30.0; *p* ≤ 0.05), and cognitive function (21.5 vs. 29.2; *p* ≤ 0.05), while three dimensions were in the normal range (physical complaints, basic motor function, and negative emotional function).
Langman et al. (2014)^17^	USA	To assess the long‐term effects of delayed‐release cysteamine bitartrate on kidney function, somatic growth, and QoL	Prospective, controlled, single‐arm study	40	17 female, 23 male Mean age: 12 years	PedsQL at every quarterly visit Change in scores over 24 months	After 24 months, persistent, significant change in patients across three subscales: social functioning (*p* ≤ 0.05), school functioning (*p* ≤ 0.01), and total function (*p* ≤ 0.05). No significant changes were seen in the other two subscales (physical and emotional).
Sadjadi et al. (2020)^18^	USA	To evaluate the feasibility of EMST and its impact on swallowing and respiratory outcome measures	Longitudinal follow‐up study	20	13 female, 7 male Age range: 20–64 years	Change in MDADI scores between two visits (1 year apart) and subsequent 5‐week exercise regimen	No significant changes in MDADI over the course of the observational phase of the study (composite scores Visit 1: 77.8 vs. Visit 2: 71.3; *p* = 0.173). No significant change in MDADI after the exercise phase (composite scores Visit 2: 71.3 vs. Visit 3: 72.8; *p* = 0.769).
Doyle and Werner‐Lin (2015)^19^	USA	To explore the experience of adults with cystinosis, and their parents, with a focus on the transition to adulthood and adult‐oriented care	Qualitative study with constant comparative method	22 patients (and 24 parents)	10 female, 12 male Age range: 18–47 years	Semi‐structured interviews and focus groups	Several elements relevant to HrQoL were identified: patients were aware that they had been spared a previously fatal disease because of changes in medicine. They recognized the impact on their general and psychosocial health. Patients sometimes viewed the outliving of their prognosis and the associated transition to adult medicine with concern, particularly concerning the continuity and quality of healthcare. Taking responsibility for their health and medical care and autonomy were also discussed.
Elenberg (2011)^16^	USA	To evaluate QoL in patients with cystinosis and compare it with their parents' QoL perception	Cross‐sectional survey	14 patients (and 14 parents)	Age range: 5–18 years	PedsQL child and parent report	Total scores calculated from patients with cystinosis were significantly higher than those of their parents (76 vs. 62; *p* ≤ 0.05). While there was no significant difference in the Physical Health domain, differences were seen in the psychosocial health domain between patients with cystinosis (higher score) and their parents (75 vs. 60; *p* ≤ 0.05). The data were compared with previously published healthy subjects and revealed a lower score for patients with cystinosis (76 vs. healthy score 87; *p* ≤ 0.01) and their parents (62 vs. healthy score 87; *p* ≤ 0.001).

Abbreviations: HrQoL, health‐related quality of life; MDADI, M.D. Anderson Dysphagia Inventory; *n*, number of subjects; *p*, *p*‐value; PedsQL, Pediatric Quality of Life Inventory; QoL, quality of life; TACQOL, The Netherlands Organization for Applied Scientific Research Academical Medical Center (TNO–AZL) Child Quality of Life Questionnaire.

### 3.3 HrQoL in patients with cystinosis

#### 3.3.1 Comparison with healthy subjects

Two of the studies identified had an observational design and aimed at describing the HrQoL of patients with cystinosis compared to healthy subjects.^10^, ^16^ In their case series study, Ulmer et al. described the child‐ and parent‐reported HrQoL of nine patients with cystinosis, aged 5–19 years, using the TACQOL.^10^ In addition to HrQoL, intellectual and motor performance and psychosocial adjustment were assessed. Compared with the reference values of healthy children, the reports of the children with cystinosis were in the normal range in six out of seven HrQoL dimensions (the exception, in this case, was the dimension of *positive emotions* (10.9 vs. 13.3; *p* ≤ 0.05)). Based on reports from their parents, HrQoL was impaired in four dimensions (*positive emotions* (11.4 vs. 15.0; *p* ≤ 0.01), *autonomy* (27.0 vs. 31.4; *p* ≤ 0.05), *social function* (26.0 vs. 30.0; *p* ≤ 0.05), and *cognitive function* (21.5 vs. 29.2; p ≤ 0.05)), while three dimensions were in the normal range (*physical complaints*, *basic motor function*, and *negative emotional function*).

Elenberg et al. measured HrQoL in 14 patients using the PedsQL cross‐sectional survey tool.^16^ The total PedsQL scores from the children with cystinosis were significantly higher than those reported by their parents (76 vs. 62; *p* ≤ 0.05). While there was no significant difference in the *physical health* dimension, differences were found between patients with cystinosis (higher score) and their parents in the *psychosocial health* dimension (75 vs. 60; *p* ≤ 0.05). Compared with previously published reports from healthy subjects, both child‐reported and parent‐reported HrQoL was significantly lower (76 vs. 87; *p* = 0.004, child‐reported HrQoL; 62 vs. 87; *p* = 0.0001, parent‐reported HrQoL).

#### 3.3.2 Effects of treatment on HrQoL

Langman et al. reported HrQoL outcomes of 40 patients with cystinosis aged 6–20 years treated with delayed‐release cysteamine bitartrate for two years.^17^ This prospective, controlled, open‐label, single‐arm study assessed HrQoL using the PedsQL at quarterly visits. At 24 months, a significant change over time was observed in the dimensions of *social functioning* (*p* ≤ 0.05), *school functioning* (*p* ≤ 0.01), and *total functioning* (*p* ≤ 0.05). No significant change was observed in the dimensions of *physical functioning* or *emotional functioning*.

In a longitudinal follow‐up study with a “pre–post” design, Sadjadi et al. evaluated the impact of a 5‐week expiratory muscle strength training (EMST) program on HrQoL and other outcomes in patients with cystinosis using the MDADI.^18^ The MDADI is a 20‐item condition‐specific PROM designed to measure the impact of dysphagia on HrQoL.^21^ Twenty patients aged 20–64 years were assessed before participating in the EMST program and then again one year later.^18^ No significant changes in dysphagia‐specific HrQoL were observed during the observational and experimental phases of the study.

#### 3.3.3 Qualitative results

A qualitative study of 22 patients aged 18–47 and their parents examined patients' experiences with cystinosis transitioning to adulthood.^19^ Notably, data collection consisted of interviews and responses to focus‐group questions rather than a proper HrQoL instrument. The themes that emerged relevant to cystinosis‐specific HrQoL included: recognition of survival despite a poor prognosis, transition to adulthood and adult medicine, adjustment of daily life activities due to cystinosis, shifting responsibility from parents to adult children, and maintaining autonomy while remaining connected to family. By surpassing their prognoses regarding health and survival, patients recognized how advances in the treatment had transformed their life expectancy and overall HrQoL, enabling them to complete school, retain jobs, engage in romantic relationships, and start families of their own. However, the transition to adulthood was associated with many concerns, particularly the ongoing need for specialized healthcare. Managing the demanding treatment regimen for cystinosis requires a high degree of organization and planning for the families and patients.^22^ Therapy‐related side effects, such as the characteristic body smell from cysteamine use, may affect treatment adherence, particularly during adolescence when social contacts outside the family and romantic relationships become more important.^19^ Some patients had concerns about taking responsibility for their treatment, given its complexity. As a result, many patients described the challenge of balancing their autonomy and continued family involvement in tasks and decisions related to their healthcare.

### 3.4 Patient‐reported outcome measures to asses HrQoL in patients with cystinosis

Our scoping review identified three PROMs used to assess HrQoL in patients with cystinosis (Table 2). The PedsQL is a generic PROM consisting of 21 respectively 23 items. The four scales focus on *physical functioning* (eight items), *emotional functioning* (five items), *social functioning* (five items), and *kindergarten* (three items), respectively *school, university, or work* (five items). This PROM is a parent‐report form for children aged 2–4 years. Both self‐report and parent‐report forms are validated for the HrQoL asessment in children (5–7 years, 8–12 years), adolescents (13–18 years), young adults (18–25 years) and adults. Cronbach's alpha ranges from 0.71 to 0.88 for the self‐report form and 0.75 to 0.90 for the parent‐report form.^23^, ^24^ The PedsQL is validated for various languages, for example, English, Dutch, French, German, Italian, Japanese, Portuguese, Russian, Spanish, Swedish, and Turkish.^25^ Additionally, the PedsQL Infant Scales focus on HrQoL in children aged 1–24 months as parent‐report forms. The Infant Scales comprises 36, respectively 45 items resulting in five dimensions (*physical functioning, physical symptoms, emotional functioning, social functioning*, and *cognitive functioning*). Cronbach's alpha ranges between 0.72 and 0.92.^26^ It is validated for several languages, for example, English, Dutch, French, German, Italian, Japanese, Romanian, Arabic, Spanish, Swedish, and Turkish.^25^

**TABLE 2:** Summary of included studies—PROMs to assess HrQoL in patients with cystinosis

PROM	Used by	Level	Age range	Languages	Scales, number of items	Cronbach's alpha
PedsQL	Langman et al. (2014)^17^ Elenberg (2011)^16^	Generic	PedsQL Infant Scales (1–24 months (PR)); PedsQL (2–4 years (PR), 5–18 years (SR and PR), 18–25 years (SR and PR), adults (SR and PR))	Arabic, Catalan (2–4 PR, 5–18 PR and SR), Cebuano (except 18–25 and adults), Croatian (except adults), Czech, Danish (except adults), Dutch, English, Estonian (8–18 PR and SR, 18–25 and adults PR and SR), Finnish (except adults), French, Georgian (2–18 PR), German, Greek, Hebrew, Hungarian, Italian, Japanese, Korean, Latvian, Lithuanian (except 18–25 and adults), Malay (except adults), Mandarin (Chinese), Norwegian (except adults), Polish, Portuguese, Romanian, Russian, Sesotho (except 13–18, 18–25, and adults), Serbian (2–4 PR, 8–12 SR, 13–18 PR and SR), Slovak (except 18–25 and adults), Slovenian (0–2 and 2–4 PR), Spanish, Swedish, Tagalog (except 18–25 and adults), Tamil (except 18–25 and adults), Thai (except 18–25 and adults), Turkish, Ukrainian, Urdu	PedsQL Infant Scales: 36 resp. 45 items resulting in 5 domains (physical functioning, physical symptoms, emotional functioning, social functioning, cognitive functioning); PedsQL: scales: 21 resp. 23 items resulting in 4 domains (physical, emotional, social, and kindergarten/school/university/work); 3 summary scores: Total Scale Score, Physical Health Summary Score, Psychosocial Health Summary Score	PedsQL Infant Scales: 0.72–0.92 PedsQL: 0.71–0.89 (5–18 years, SR); 0.74–0.92 (2–18 years, PR)
TACQOL	Ulmer et al. (2009)^10^	Generic	TAPQOL (PR, 9 months −5 years); TACQOL‐PF 6–15 (PR); TACQOL‐CF 8–15 (SR); TAAQOL (SR, 16+ years)	TAPQOL: Bulgarian, Chinese, Croatian, Dutch, English, French, German, Italian, Korean, Portuguese, Romanian, Russian, Spanish, Turkish, Vietnamese TACQOL: Bulgarian, Chinese (only SR), Dutch, English, French, German, Italian, Korean, Russian, Spanish, Vietnamese (only SR) TAAQOL: Chinese, Dutch, English, German	TAPQOL: 32 or 43 items in 9 scales (stomach problems, skin problems, sleeping problems, appetite, problem behavior, positive mood, liveliness) TACQOL: 56 items in 7 scales (8 items per scale: physical, motor functioning, autonomy, cognitive functioning and school, social contacts, positive emotions, and negative emotions) TAAQOL: 45 items in 12 domains (gross motor functioning, fine motor functioning, cognition, sleep, pain, social contacts, daily activities, sex, vitality, happiness, depressive mood, and anger)	TAPQOL: 0.43–0.84 (PR) TACQOL: 0.65–0.79 (SR); 0.67–0.84 (PR) TAAQOL: 0.72–0.90 (SR)
MDADI	Sadjadi et al. (2020)^18^	Disease‐specific (dysphagia)	Adult patients	Arabic, Chinese, Danish, English, French, German, Indonesian, Italian, Japanese, Portuguese, Swedish, Spanish	4 scales (global 1 item, emotional 6 items, functional 5 items, physical 8 items)	0.85–0.93

Abbreviations: MDADI, Monroe Dunaway Anderson Dysphagia Inventory; PedsQL, Pediatric Quality of Life Inventory; PR, parent‐report; PROM, patient‐reported outcome measurement; SR, self‐report; TAAQOL, TNO‐AZL Questionnaire for Adult's Health related Quality of Life; TACQOL–CF, TNO‐AZL Children's Quality of Life Questionnaire‐Child Form; TACQOL‐PF, TNO‐AZL Children's Quality of Life Questionnaire‐Parent Form; TAPQOL, TNO‐AZL Preschool children Quality of Life Questionnaire; TNO‐AZL, Preschool children Quality of Life Questionairre; TNO‐AZL, toegepast‐natuurwetenschappelijk onderzoek van het Academisch Ziekenhuis in Leiden.

The TACQOL is a generic PROM to assess HrQoL in healthy children and children with different chronic diseases aged 6–15 years. Parent‐report forms are validated for children aged 6–11 years, and self‐report forms are available for children aged 8–15. The questionnaire consists of 56 items, including seven scales with eight items for each scale (*physical, motor functioning, autonomy, cognitive functioning and school, social contacts, positive emotions*, and *negative emotions*). Cronbach's alpha varies between 0.65 and 0.79 for the self‐report form and between 0.67 and 0.84 for the parent‐report form.^27^ The TACQOL is validated for, for example, Bulgarian, Dutch, English, French, German, Italian, Russian, and Spanish.^27^

There are also validated versions of this tool for preschool children (TAPQOL) aged 9 months up to 6 years and adults aged 16 years and above (TAAQOL). The TAPQOL consists of 32 respectively 43 items resulting in nine scales (*stomach problems, skin problems, sleeping problems, appetite, problem behavior, positive mood*, and *liveliness*) respectively 12 (plus *social functioning, motor functioning*, and *communication*), depending if the children's age is more than 18 months. Cronbach's alpha for a general population sample varies between 0.43 and 0.84.^28^ The preschooler's version is validated for, for example, Bulgarian, Chinese, Dutch, English, French, German, Italian, Korean, Romanian, Russian, Spanish, and Vietnamese.^28^, ^29^ The TAAQOL comprises 45 items covering 12 domains (*gross motor functioning, fine motor functioning, cognition, sleep, pain, social contacts, daily activities, sex, vitality, happiness, depressive mood*, and *anger*). Reliability ranges between 0.72 and 0.90.^30^ The TAAQOL is validated in Dutch, English, and German.^31^

The Monroe Dunaway Anderson Dysphagia Inventory (MDADI) is a disease‐specific PROM focusing on dysphagia in adult patients. The four scales of the MDADI include *emotional*, *functional*, and *physical* aspects of HrQoL as well as one *global* item. The reliability varies between 0.85 and 0.93.^21^ The MDADI is validated for, for example, Arabic,^32^ Chinese,^33^ Danish,^34^ English, French,^35^ German,^36^ Indonesian,^37^ Italian,^38^ Japanese,^39^ Portuguese,^40^ Swedish,^41^ and Spanish.^42^

## 4 DISCUSSION

This scoping review identified five articles describing HrQoL in patients with cystinosis.^10^, ^16^, ^17^, ^18^, ^19^ While two studies compared HrQoL scores of patients with cystinosis with those of healthy subjects,^10^, ^16^ two studies addressed the change in HrQoL resulting from new treatment procedures.^17^, ^18^ The fifth study described the transition experience to adulthood, with important HrQoL implications.^19^ Although data are limited, with few dedicated studies and small sample sizes, these results suggest that cystinosis has a negative impact on HrQoL, with the most significant impact being observed in the psychosocial dimension of HrQoL. This is consistent with observations from studies of patients with cystinosis that have focused purely on psychosocial effects (and therefore were omitted here).^43^, ^44^

Our review shows that research on HrQoL in patients with cystinosis is still rare. To fully understand the impact of cystinosis on HrQoL, additional observational studies with larger sample sizes and longitudinal studies are needed. In this context, it is also important to clarify which domains of life are affected by the disease and to what extent adaptation to the disease and treatment side effects come into play.

Validated PROMs are suitable measurement tools, particularly for the characterization of HrQoL. The three quantitative studies in our review used PROMs to assess HrQoL^10^, ^16^, ^17^, ^18^: two generic PROMS (PedsQL, TACQOL), while one was condition‐specific (MDADI), focusing on dysphagia. It remains unclear whether or not the MDADI has been validated for use in patients with cystinosis; this is an essential prerequisite for the successful future use of such an instrument.^45^

Generic, chronic‐generic, and condition‐specific PROMs have unique advantages and disadvantages that should be considered depending on their intended use.^12^ Generic PROMs enable comparisons with the general population, while chronic–generic PROMs enable comparisons with populations affected by other health conditions.^46^ For reporting purposes and a general understanding of the disease through comparisons with, for example, country‐specific reference values, generic and chronic–generic PROMs represent a good choice. In both descriptive studies, it was noticeable that the HrQoL ratings of children with cystinosis were higher than the ratings of their parents.^10^, ^16^ Differences in child‐reported and parent‐reported HrQoL have also been reported in other studies of rare pediatric diseases.^47^ Thus, including child self‐ratings and parent proxy ratings remains essential to allow a balanced presentation.

In clinical trials, condition‐specific PROMs could offer important advantages. Specific PROMs can be particularly helpful for rare chronic health conditions as they address the most specific concerns of unique patient populations (including treatment‐related concerns, such as side effects) and are more sensitive to condition‐specific changes.^46^ However, in the studies screened, which comprised numerous clinical trials, we found that HrQoL has played a relatively minor role in assessing new therapies for cystinosis compared with the more traditional clinical parameters and clinical outcomes. Including PROMs, particularly HrQoL instruments could support further clinical research in cystinosis, with an additional focus on reimbursement.^48^

Currently, there is no cystinosis‐specific instrument for measuring HrQoL in this patient population; qualitative research is needed to develop a cystinosis‐specific HrQoL PROM. In the meantime, allowing patients to share their stories, makes it possible to understand their experiences in detail, capture the full spectrum of manifestations, and identify any possible relationships with HrQoL.

In the one qualitative study included in this review, valuable insights emerged from the focus group discussions and interviews, such as how treatment progress has opened up new life perspectives and milestones in the affected individuals' lives or how side effects have impacted social relationships and consequently treatment adherence and health outcomes.^19^ Such insights are crucial for characterizing condition‐specific HrQoL. The qualitative results from this study refer exclusively to patient experiences during their transition to adulthood, which is essential given that medical advances have made this possible. Overall, qualitative insights into the HrQoL of children with cystinosis are lacking, and future research should aim to investigate this in more detail.

When developing and validating a cystinosis‐specific HrQoL instrument, the recommendations on patient‐reported outcome measurements provided by the International Society for Pharmacoeconomics and Outcomes Research and US Food and Drug Administration need to be considered and have to include all steps; literature review, focus interviews with patients and/or parents, development of a pilot‐test, field‐ and re‐test as well as linguistic and cultural validation for use in additional languages.^45^, ^49^, ^50^, ^51^, ^52^ Clinical parameters and age‐specific aspects must be considered in developing a condition‐specific PROM to ensure that it is sensitive to the severity of the disease or symptoms and changes due to treatment. In addition, it is necessary—especially in pediatrics—to include patient‐reports and proxyreports by, for example, parents.^53^ The rarity of the disease presents challenges for developing and validating a PROM. While costly international studies may increase the sample size required for validation, language, and cultural differences must also be considered.^49^

The experience of the cross‐cultural development, validation, and cultural adaptation of condition‐specific PROMs such as the Quality of Life in Short Stature Youth (QoLISSY) questionnaire to assess the HrQoL of short stature children and adolescents^54^, ^55^, ^56^, ^57^ or the EA‐QoL questionnaire for children and adolescents with esophageal atresia^58^, ^59^, ^60^, ^61^, ^62^ should be considered. Identifying disease‐specific PROMs covering symptoms that may occur in patients with cystinosis, such as dysphagia,^63^ could be a starting point for developing a PROM.

Our scoping review did not identify any study based on registries, although we included registries due to the a prior‐defined inclusion criteria. For validation of a condition‐specific PROM, registries have an enormous potential to assess HrQoL and to monitor HrQoL regarding progression and treatment. Regular use of validated PROMs in registries is desirable. It requires a good collaboration of experts in quality of life research, clinical care, and patient organization to provide the best possible care that considers the patient's perspective.

The small sample sizes and the fact that four out of five studies were conducted in the United States, while one was from Switzerland, must be considered when interpreting the results and highlighting the need for further research. The present review does not claim to identify every existing study on HrQoL in patients with cystinosis. Instead, this scoping review has allowed us to summarize research findings, make conclusions about the general state of research, and highlight any research gaps. In addition, it is essential to emphasize that quality assessments are not part of a scoping review.^13^

In summary, few studies have investigated HrQoL in patients with cystinosis. A few studies suggest that HrQoL is reduced in patients with cystinosis compared with healthy individuals. Additional observational studies describing HrQoL using mixed methods with quantitative and qualitative elements and an equal focus on all age groups are needed. Especially in rare diseases, data must be collected standardized and follow the FAIR (Findable, Accessible, Interoperable, and Reusable) principles.^64^ The use of validated generic and condition‐specific HrQoL instruments can contribute to a deeper understanding of the impact of cystinosis on patients and also support ongoing clinical research.

## AUTHOR CONTRIBUTIONS

Kaja Kristensen, Stefanie Witt, Katharina Hohenfellner, and Julia Quitmann developed the study concept and the design. Kaja Kristensen and Stefanie Witt screened the publications and identified relevant articles. Kaja Kristensen wrote the first draft of the manuscript. Stefanie Witt, Julia Quitmann, and Katharina Hohenfellner critically reviewed and revised the first draft for important intellectual content. All authors have critically revised subsequent drafts of the manuscript. All authors read and approved the final manuscript to be published and agreed to be accountable for all aspects of the work.

## FUNDING INFORMATION

This scoping review was funded by Cystinosis Foundation Germany. The sponsor has not influenced the content of the article.

## CONFLICT OF INTEREST

Stefanie Witt, Kaja Kristensen, Katharina Hohenfellner, and Julia Quitmann have no competing interests.

## ETHICS STATEMENT

Not applicable.

## A PATIENT CONSENT STATEMENT

Not applicable.

## DOCUMENTATION OF APPROVAL FROM THE INSTITUTIONAL COMMITTEE FOR CARE AND USE OF LABORATORY ANIMALS (OR COMPARABLE COMMITTEE)

Not applicable.

## Data Availability

Data sharing is not applicable to this article as no new data were created or analyzed in this study.
